# A Bayesian framework to evaluate non-inferiority in randomized controlled trials of uncommon conditions

**DOI:** 10.1016/j.jclinepi.2025.112002

**Published:** 2025-09-29

**Authors:** Victoria R. Cornelius, Jack Elkes, Ian R. White, Rebecca M. Turner, Michelle Clements, Matteo Quartango, Conor D. Tweed, Sejal Saglani, Suzie Cro

**Affiliations:** a Imperial Clinical Trials Unit, School of Public Health, Imperial College London, London, UK; b MRC Clinical Trials Unit at UCL, 90 High Holborn, London, UK; c National Heart & Lung Institute, Imperial College London, Exhibition Road, London, UK

**Keywords:** Bayesian, Noninferiority, Randomized controlled trial, Uncommon conditions, Design, Restricted sample size

## Abstract

**Background and Objectives:**

Noninferiority (NI) trials typically require larger sample sizes than superiority comparisons. This is problematic for uncommon conditions where recruitment is restricted. When a power calculation results in an unfeasibly large sample size, there is a need to justify whether a trial in an uncommon condition is worth undertaking. We propose reversing the question to demonstrate what can be shown with a feasible maximum sample size using a Bayesian framework in place of a traditional power calculation.

**Methods:**

We propose using the posterior probability of noninferiority to demonstrate the value of undertaking an NI trial using a credible NI margin. We describe a five-part Bayesian framework: the data generation/analysis model; maximum feasible sample size; different potential outcomes; primary NI margin; and plausible priors. We illustrate the framework in an NIHR-funded NI trial of mepolizumab compared to omalizumab in children with severe therapy resistant asthma. We examine the trial operating characteristics when mepolizumab is inferior, identical, and superior to omalizumab under four differing prior distributions on the treatment effect (3 informative, 1 vague) and demonstrate suitable interpretation.

**Results:**

Our case study had a maximum feasible sample size of 150 severe therapy resistant asthmatic children. Using the proposed Bayesian framework we demonstrated that if mepolizumab was truly identical or superior to omalizumab, then the average posterior probability of noninferiority would be reassuringly high, from 0.87 to >0.99. The probabilities were reassuringly low when mepolizumab was inferior (≤0.22). The framework provided a comprehensible summary for reviewers to judge the value of undertaking the trial.

**Conclusion:**

A Bayesian approach using posterior probability for NI trials can offer a practical way to assess the value of undertaking a trial in an uncommon condition with a fixed sample size as an alternative to a power calculation.

## 1 Introduction

Noninferiority (NI) trials are designed to demonstrate no important loss of effect with the new treatment compared to an established treatment. The aim is to examine whether the new treatment is not worse than the established treatment by an acceptable margin. This is suitable when the new treatment offers other advantages such as fewer side effects or lower cost [1,2]. A prespecified NI margin (δ^∗^) is selected to represent the amount judged to be the smallest loss of effect, relative to the established treatment, that is considered to be clinically unacceptable. The aim is to determine that the treatment effect (δ) is less than the non-inferiority margin by testing the null hypothesis δ ≥ δ^∗^ against the alternative hypothesis δ>δ^∗^, where lower values of δ are more favorable. Sample size for a noninferiority trial is highly dependent on the chosen size of δ^∗^, with smaller δ^∗^ requiring larger sample size when the outcome value is assumed to be the same in both arms. In the analysis, if the whole 95% confidence interval for the treatment effect lies below δ^∗^, noninferiority can be declared.

The sample size for an NI trial is often larger than that needed for a superiority trial, as the NI margin is usually smaller than the difference used for powering a superiority trial [3]. This can be particularly challenging when evaluating treatments in uncommon conditions (rare diseases or specialist subgroups of more common conditions). Parmar et al proposed a practical framework for sizing trials using a significance-based hypothesis testing approach for small populations [4]. While it mainly assumes superiority comparisons, many aspects apply to the NI settings. However, some approaches to reducing sample size, such as relaxing the significance level, do not translate well to NI comparisons.

Selecting a single NI margin, which should combine statistical and clinical reasoning, can be difficult [5]. The NI margin may vary by stakeholder (policymakers, prescribers, and patients) [6]. This challenge is made harder when the outcome is binary, such as mortality or morbidity events, as deciding on the minimum number of “extra” events that are ethically unacceptable is subjective [1]. In practice, trials sometimes use wide NI margins without justification [7–10], raising concerns whether the driving factor has been the number thought feasible to recruit. This opens the trial to harmful conclusions. In a frequentist setting the δ^∗^ chosen is pivotal to inference as if it does not truly represent the smallest loss of effect considered to be clinically unacceptable, then there is a risk of falsely declaring noninferiority when an important loss of effect does exist.

A Bayesian framework for the design and analysis of late phase trials offers a different philosophical approach. For our context it can provide [11]:

1) **Efficiency in the analysis by incorporating existing information for the treatment effect through the prior distribution** [12]. In general, the more informative the prior, the greater the influence on the posterior distribution. A vague prior will add no benefit over a frequentist analysis in terms of increasing efficiency.
2) **A posterior distribution for the treatment effect that can be used to make probability statements different context for interpretation**. Probability for non-inferiority can be more intuitive to understand than *P* values and confidence intervals [13]. It encourages assessment of the strength of evidence and discourage focus for categorizing results into significant/not-significant. Analysis using confidence level and clinical significance curves across differing NI margins may similarly be performed in a frequentist framework [14], but they are rarely used.
3) **Evaluation of differing noninferiority margins that vary by stakeholder**. Probabilities can be interpreted without reference to an arbitrary threshold [15].

While Bayesian designs are often used in early-phase studies for dose-finding and decision-making, their adoption in late-phase trials is slower. Two major barriers are insufficient knowledge of Bayesian methods and lack of regulatory clarity, particularly concerning Type I error control [16]. The need for alternative to significance-based hypothesis testing approach is well recognized [17–20] and a Bayesian approach offers a valuable solution [21–23].

We propose a general framework that can be used by those designing trials to demonstrate the value of undertaking an NI trial where the sample size is highly restricted without compromising on a credible NI margin.

## 2 Methods

We use a Bayesian framework to demonstrate what evidence can be gained from an NI trial in an uncommon condition as an alternative to a frequentist power calculation. We propose how to develop and present simulation results in a concise and accessible way to facilitate review.

Our primary interest is to obtain a posterior probability distribution for three realistic potential treatment effects.

**Figure 1:**
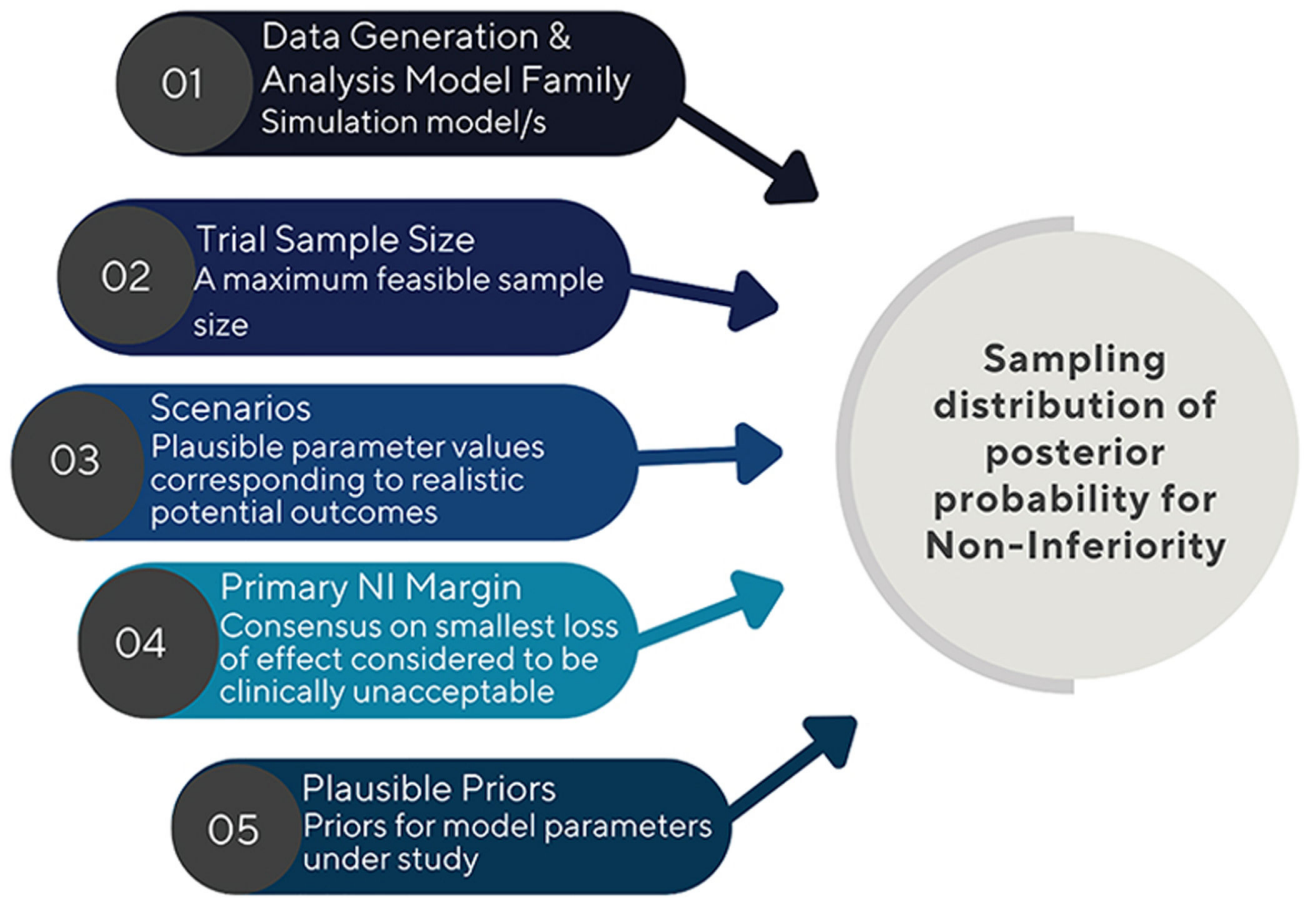
Framework to demonstrate the value of a noninferiority trial in an uncommon condition using a Bayesian analysis.

The five key inputs required are outlined in Figure 1 and introduced below. We propose restricting the number of potential treatment effects and plausible priors to ensure results are comprehensible to reviewers. In our example we use 12 scenarios (3 potential treatment effects x 4 priors). For each simulated dataset the posterior probability distribution for the treatment effect is derived. A metric, such as the average posterior probability of noninferiority is then presented. The five key inputs are.

1) **Data Generation and Analysis Model Family:** *Specify the model to be used, eg, Poisson or Normal model, to generate and analyze the data. The model parameters are not yet specified at this point*.
2) **Trial Sample Size:** *Specify the trial’s “maximum sample size” which is feasible to recruit across an extensive network of sites within a fundable time frame as recommended in Parmar et al*. [4].
3) **Scenarios:** *Specify plausible parameter values to be used in generating the data from the model specified in 1. We suggest limiting to three realistic potential treatment effects, for example, 1) both treatments have similar benefit; 2) the new treatment is superior by a justifiable size; 3) the new treatment is truly inferior by a justifiable size*.
4) **Primary Noninferiority (NI) Margin:** *Select a primary NI margin that represents the smallest loss of effect that is considered to be clinically unacceptable. Note that while we propose selecting a primary NI margin, we endorse presenting a NI margin curve or undertaking an acceptability curve estimation in the final analysis*. [15].
5) **Plausible Priors:** *Specify the prior distribution(s) for the final analysis. Before obtaining funding, the main prior for the treatment effect may be unknown. We suggest examining four alternative priors, three informative (optimistic (in favor of new treatment), skeptical (in favor of old treatment), and no-difference (not favoring either) and one vague. For simplicity, we use the terminology for the priors in reference to the treatment effect, rather than the hypothesis we are examining, that is, noninferiority, so the no-difference prior is optimistic for NI hypothesis*.

In the absence of other information, we suggest selecting the same parametric distribution for all prior distributions but with varied locations. Note that these priors are for the design stage only and will not usually be used in the analysis. A sensitivity analysis around the prior in the final analysis is a separate consideration. Choosing the parameters that define the distribution requires judgment. They can be chosen on what is reasonable to expect based on existing knowledge. Since these are for “stress testing” potential scenarios, approximate evaluations are acceptable. Prior distributions for other model parameters (such as randomization stratification variables) can remain vague in simulations if no prior information is available.

Using 1—5, for each scenario, in each simulated dataset, we can then obtain a posterior distribution to calculate the probability for NI for each of the scenarios. Different deterministic and approximate approaches, such as integrated nested Laplace approximations, can be used to calculate the posterior distribution depending on and preferences of researchers [24]. We use a readily accessible approach of Markov Chain Monte Carlo (MCMC) sampling, selecting a burn-in period, MCMC sample size, and thinning to reduce autocorrelation.

We then calculate the posterior probability of the new treatment being noninferior to the existing treatment (ie, Pr(δ<δ*| *data*), see Supplementary Fig 1). Next, we obtain a summary statistic, such as the average posterior probability of the treatment effect being < NI margin across simulations, that is,

$$\frac{1}{n_{sim}}\sum_{i=1}^{n_{sim}}\text{Pr}\left(\delta <\delta^{*}|data_{i}\right)$$

where *n*_*sim*_ is the total number of simulations, δ is the treatment effect and δ^∗^ is the noninferiority margin.

These results are then presented to reviewers to assess the value of the trial. We hope to see average posterior probabilities of noninferiority are high when the experimental treatment is truly noninferior or superior, and reassuringly low when the experimental treatment is truly inferior. The probability should be judged in context as to whether it provide “convincing” evidence for the trial to influence clinical practice. Table 1 outlines a proposed simulation set.

## 3 Case study

The treating severe pediatric asthma; a randomized controlled trial of mepolizumab and omalizumab (TREAT) aimed to examine the noninferiority of mepolizumab (new) to omalizumab (established) on asthma exacerbation rate over 12 months [25]. This trial aimed to recruit a rare sub-group of children who suffer severe asthma despite taking optimal therapy. We estimated that it would be feasible to recruit around 150 children over 3—4 years across an extended network of specialist sites nationally. Based on previous studies in this population we expect that we will obtain full outcome data on 130 children [26]. We applied the framework to demonstrate to reviewers the value of undertaking a trial of this size for a funding application. Assuming 15% loss to follow-up, 130 children would provide outcome data in the final analysis. The five inputs for TREAT can be seen in Table 2 and a description of how these inputs were selected can be found in Supplementary File 1. Example Stata code for one scenario can be found in Supplementary File 2.

For MCMC sampling we used a burn-in of 1000 followed by 80,000 simulations with thinning of every 4 resulting in 20,000 simulations. For each of the 12 scenarios we generated 1000 simulated trials. As overdis-persion in the outcome is possible we also examined a negative binomial regression model for the analysis model. Data for overdispersion in simulations were generated by drawing observations from a Poisson distribution with appropriate mean value and adding a random draw from a gamma distribution using a shape parameter of 1 and a scale parameter of 0.6 (estimated from clinical audit data).

The scenarios in Table 1 enable us to examine prior distributions that contrast and agree with the treatment effect. A Poisson regression model with offset for time was used to compare 12-month asthma exacerbation rate between arms, with Gaussian prior for δ (treatment effect, between-arm change in log exacerbation rate). The model and justifications for design parameters are reported in Supplementary File 1.

## 4 Results

For the TREAT trial in a frequentist setting, assuming an event rate of 2.5 exacerbations per year in the both groups, a noninferiority margin of 0.5 exacerbations per year, a 15% loss to follow-up, using one-sided 0.025 type I error, we would need to recruit around 600 participants to achieve 90% power. Allowing for potential overdispersion in the outcome using a negative binomial model (0.6 scale parameter estimated from audit data), would increase this to 1490 participants. This made the trial unfeasible in a frequentist framework as recruitment would take between 12 and 26 years.

We applied the Bayesian framework to calculate average posterior probabilities for 12 simulation scenarios. We assumed potential treatment effects for mepolizumab to be: better by 1 exacerbation per year; no different; or worse by 1 exacerbation per year. The results demonstrate that if mepolizumab is truly equal or superior to omalizumab (scenarios 1—8) then the trial has the potential for providing high posterior probabilities of noninferiority, with average posterior probability ranging from 0.87 up to >0.99, depending on whether vague or informative priors are used (Table 3). Probabilities this high would likely provide reassurance that a trial of this size has the potential to inform treatment decisions. The table can be presented in its entirety in place of a frequentist sample size calculation, along with clear justifications for parameter choices for the assumed scenarios and prior distributions.

**Figure 2:**
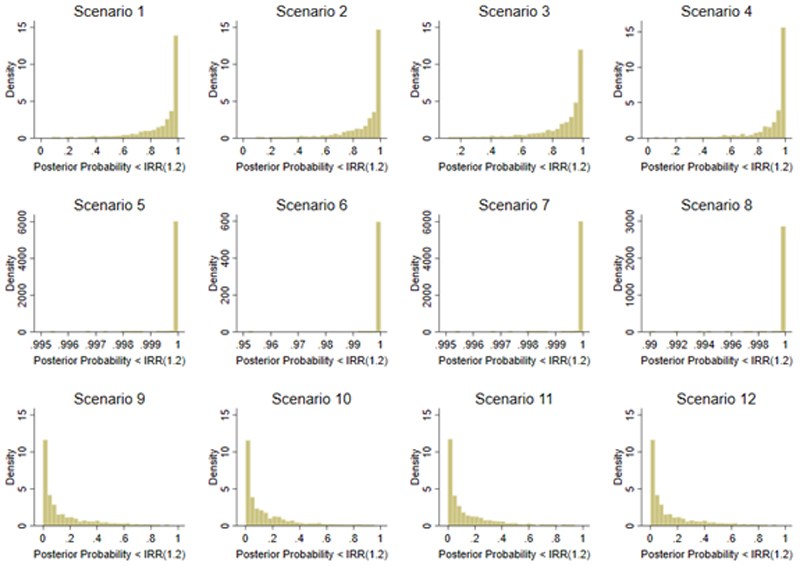
Posterior probabilities for noninferiority (margin 0.5) from 1000 trial simulations when sample size is 130 across each of the 12 scenarios explored for the TREAT case study. TREAT, treating severe pediatric asthma; a randomized controlled trial of mepolizumab and omalizumab.

As the table only provides the average posterior probability across the simulations, a measure of spread (IQR, SD) can be added, or a histogram presented. Figure 2 displays the distribution of the posterior probabilities for each scenario and shows when there is no meaningful loss in efficacy with mepolizumab (scenarios 1—4), posterior probabilities for noninferiority will be high and predominantly above 0.8. When mepolizumab is superior to omalizumab, posterior probabilities for noninferiority will always be above 0.99, while when mepolizumab is inferior, posterior probabilities are predominantly <0.3.

A natural curiosity is the impact of a smaller sample size. We repeated the simulations for 75% of the chosen maximum sample size. The results in Table 3 show lower average posterior probabilities ranging from 0.80 to 0.89 when mepolizumab was the same as omalizumab. This suggests a smaller sample size could still provide evidence strong enough to inform prescribing decisions. There will always be value in adding participants to a trial, examining a smaller sample size provides perspective but we do not suggest this information should be used to inform a reduction in sample size. Whether one would want to reduce the sample size is a complex issue and requires trading off gains in precision against increased resources required. This entails careful consideration and is addressed in Turner et al [27].

The prior distributions to be used in the TREAT final analysis are unknown and will be obtained through a clinician-elicited behavioral aggregation approach, constructing consensus prior distributions based on experts’ evaluation of published data [28]. We examined the impact of a range of prior distributions in the simulations and found these to be relatively unimportant. See Supplementary Figure 2 that depicts the posterior mean and 95% Cred. Int. by scenario showing the influence of the priors for TREAT. On one hand this is reassuring for reviewers skeptical of incorporating prior information due to concern around subjectivity, though this does mean that one of the potential advantages of using a Bayesian analysis (increasing the precision of the treatment effect estimate) may not be fully realized for this study unless the sample size is smaller and the outcome in over dispersed. This result is based on assumptions for plausible informative and skeptical priors, and in practice our choices may have been too conservative.

When mepolizumab was inferior to omalizumab the average posterior probabilities were reassuringly low, 0.14 for 100% sample size, and 0.16—0.21 for 75% sample size. Note that these probabilities should not be compared to typical type 1 error rate of 0.05 used in a frequentist setting as they are not the same. The question is whether, given these low probabilities, a decision would be made to incorrectly change prescribing or policy recommendations to endorse mepolizumab. With such low probabilities this seems unlikely.

We also examined overdispersion in the outcome and found lower probabilities would be obtained, with means of 0.71 to 0.83 if mepolizumab was equally effective to omalizumab (Table 3). These probabilities now arguably start to include a range where the trial result could be considered less convincing for influencing practice. Whether clinicians and policy makers would change prescribing based on these probabilities needs further consideration and consultation from the community. The other scenarios for superiority and inferiority still provided clear cut convincing probabilities to aid decision-making with >0.97 when mepolizumab was superior and <0.44 when mepolizumab was inferior.

## 5 Discussion

Restricted sample size presents a significant design challenge for clinical trials in uncommon conditions. Stake-holders need to assess the value of a trial when traditional power calculations with realistic NI margin assumptions aren’t possible. A trial’s value lies in providing evidence to inform treatment recommendations. We propose a practical design approach to improve the efficiency of noninferiority trials in rare conditions, which can also be applied to superiority trials, while not compromising on the width of the NI margin. This prevents invalid inferences and potentially harmful conclusions being made in a frequentist framework.

Sample size may also be restricted due to extremely high treatment costs or availability of a novel intervention. These reasons should be differentiated from occasions when trialists feel unable to recruit due to lack of funding or network limitations. Regardless of the reasons for a restricted sample size, a well-considered design and analysis approach is essential to draw appropriate conclusions.

The rare disease community advocates use of adaptive design features, Bayesian methods, and n-of-1 trials as a way to overcome limited sample size [29]. Approaches for sample size calculations include calculating assurance and use of a decision-theoretic approach. Miller et al describe these both clearly with applications demonstrated for three rare disease trials [30]. There are many approaches to increase statistical efficiency within significance-based hypothesis testing framework that can reduce the required sample size. Classic design features include the use of crossover designs, repeated measurements and use of information rich outcomes. Parmar et al outline these and other key aspects in a practical framework to guide investigators through the sizing of a trial which should also be fully explored [4]. While these aspects were originally explored in the TREAT trial, they were not accepted by the funders as a suitable solution.

If the simulation finds high posterior probabilities of NI when the treatment is effective, this provides reassurance to reviewers that there is value in undertaking the trial. When probabilities are midrange (eg, 0.55—0.7) this judgment will be less clear cut. Ideally a pre-enquiry of stakeholders for the probability range that would result in them recommending/accepting the treatment would be desirable to inform inference. This could take place during the protocol development phase. It could include a formal survey or consensus elicitation of the target stakeholders. Stakeholders will depend on the intervention being evaluated, for example, this maybe treating clinicians, or commissioners if requiring the availability of new treatments. Patient and public perspective could also be included, and be an additional component of the Patient and Public Involvement and Engagement (PPIE) activities within a trial to undertake this component.

While the simulations are based on the primary NI margin, it is worth noting that we do not need to limit ourselves to a single NI margin for the final Bayesian analysis, as would be required in a frequentist evaluation. As the Bayesian framework allows direct interpretation of the treatment effect in terms of probabilities we can examine a range of NI margins, which improves transparency if the NI margins differ by stakeholder [15]. The problem of using a single fixed NI margin has also been examined by other researchers for prespecified binary safety outcomes that require interim monitoring in a trial. Aupiais et al proposes use of a Bayesian analysis to develop thresholds to guide decision-making during safety monitoring, where the NI Margin has been informed by a formal elicitation from experts [31]. Their approach means that the experts’ uncertainty around the NI margin is incorporated.

A Bayesian approach to noninferiority trials is not limited to uncommon settings. We would recommend its use in common disease settings for its improved interpretation, the transparency it can provide around presenting results for varying noninferiority margin, and its ability to incorporate existing information. In a common disease setting we would advocate a different approach to sizing the trial, using a Bayesian power based approach or calculating the expected posterior probability as detailed in Turner et al [27].

In the same way that frequentist sample size calculation can be vulnerable to an overoptimistic NI margin or minimally clinically important difference, the Bayesian approach has the same vulnerability. It is important that reviewers scrutinize the simulation assumptions, in particular to ensure that the potential outcomes are not overoptimistic and the location and precision of the prior distributions look, and the primary noninferiority margin is not too large.

Trials with highly restricted sample sizes are extremely challenging to design. In the frequentist setting, unrealistic sample size assumptions, especially for the minimum clinically important difference, often lead to invalid inference [32]. A Bayesian framework offers a valuable solution for late phase trials in uncommon conditions. We have demonstrated designing an NI trial using a Bayesian approach for restricted sample sizes and proposed a framework to aid re-viewers in evaluating the trial.

## Supplementary Material

Supplementary data related to this article can be found at https://doi.org/10.1016/j.jclinepi.2025.112002.

Supplementary File 1

Supplementary File 2

## Figures and Tables

**Visual Abstract:**
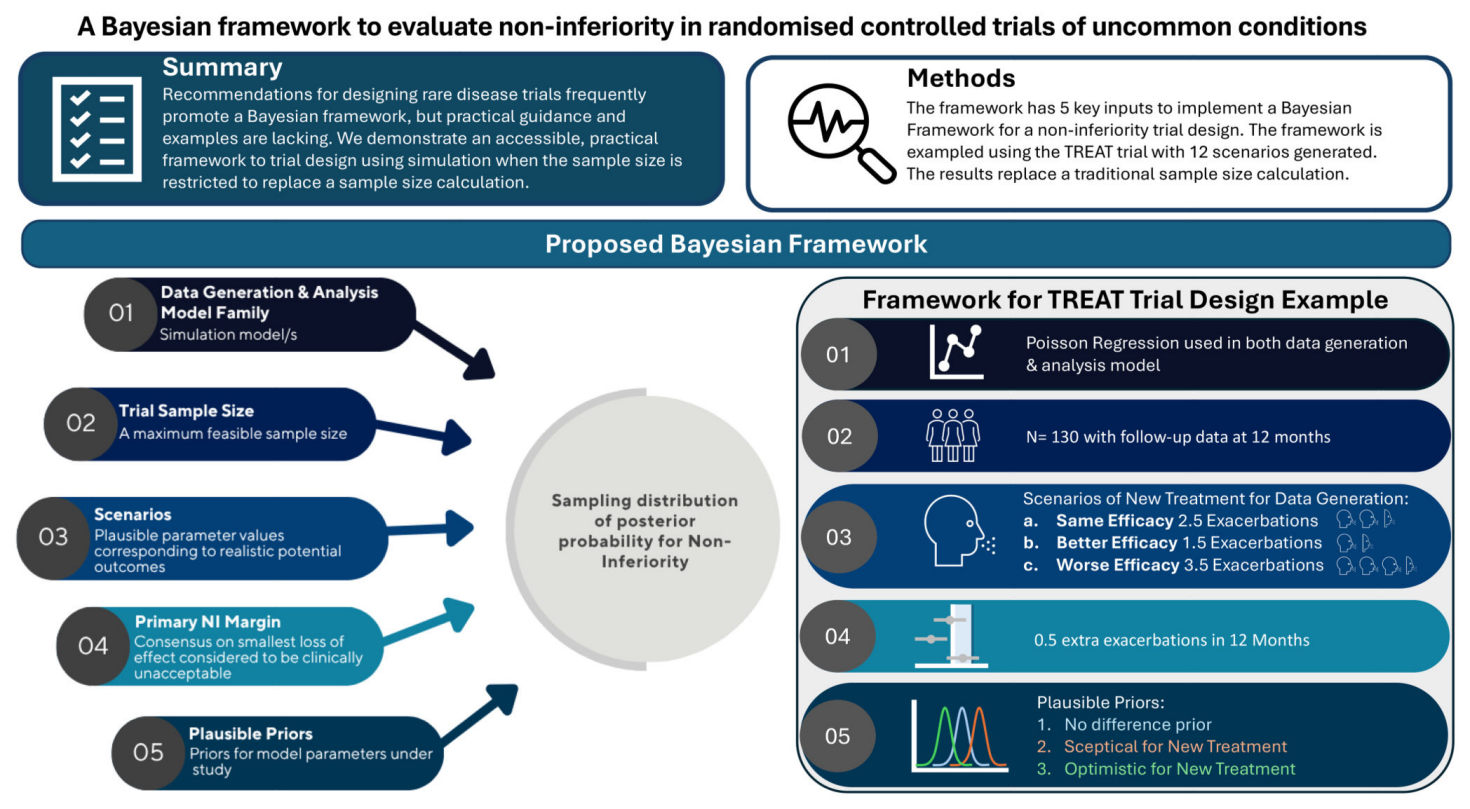


**Table 1:** The simulation set― 12 scenarios to examine the potential contribution of the trial to the evidence base

Treatment effect examined	Prior distribution on the treatment effect (*δ*)	Prior on the treatment effect	Scenario
New treatment is the same as established	Vague	NA	1
	New is the same established	No difference prior	2
	New is worse than established	Skeptical prior	3
	New is better than established	Optimistic prior	4
New treatment is better	Vague	NA	5
	New is the same established	No difference prior	6
	New is worse than established	Skeptical prior	7
	New is better than established	Optimistic prior	8
New treatment is worse than established	Vague	NA	9
	New is the same as the established	No difference prior	10
	New is worse than established	Skeptical prior	11
	New is better than established	Optimistic prior	12

NA, not applicable. n.b. “new” is the new treatment being evaluated, “established” is the existing treatment that noninferiority is being examined against.

**Table 2:** The five inputs for the Bayesian framework for the TREAT trial design

(1) Data generation and analysis model family	Poisson regression model with an offset for time log(μ_i_) = *α* + *δ* x_i_ + log(t_i_) *α*: log exacerbation rate in omalizumab arm *δ*: difference in log exacerbation rate between arms
(2) Trial sample size	*N =* 150 with 130 having full follow-up data at 12M
(3) Scenarios	a. Mepolizumab the same efficacy as omalizumab, both with mean 12M exacerbation rate of 2.5 b. Mepolizumab better efficacy than omalizumab, with mean 12M exacerbation rate of 1.5 c. Mepolizumab inferior efficacy to omalizumab, with to mean 12M exacerbation rate of 3.5
(4) Primary noninferiority (NI) margin	NI margin of 0.5 exacerbations per 12M
(5) Plausible priors	Informative prior for *α* ~ N(0.916, 0.347)//used for all scenarios Vague prior for *δ* ~ N(0.0, 100) No difference prior for *δ* ~ N(0.0, 0.347) Skeptical for mepolizumab prior *δ* ~ N(0.34, 0.347) Optimistic for mepolizumab prior *δ* ~ N(-0.51, 0.347) See Supplementary Figure 1 for a graphical depiction of the prior distributions used


TREAT, treating severe pediatric asthma; a randomized controlled trial of mepolizumab and omalizumab. Nb. Normal prior parameters are N (mean, SD).

**Table 3:** Posterior probabilities for noninferiority for 100% and 75% of the recruitable sample, using both Poisson regression and negative binomial regression

Treatment effect^†^	δ Gaussian prior Mean, SD (type)	Scenario	Average probability of noninferiority when *n* = 130		Average probability of noninferiority when *n* = 98		Average probability of noninferiority when *n* = 130		Average probability of noninferiority when *n* = 98
			(100% sample size)		(75% sample size)		(100% sample size)		(75% sample size)
			Poisson distribution for outcome (primary)				Negative binomial distribution for outcome		
No Difference (omalizumab = 2.5)– (mepolizumab = 2.5) = 0	0.00, 100 (vague)	1	0.88		0.8		0.72		0.69
	0.00, 0.347 (no difference)	2	0.89		0.83		0.77		0.76
	0.34, 0.347 (skeptical for mepolizumab)	3	0.87		0.83		0.71		0.7
	– 0.51, 0.47 (optimistic for mepolizumab)	4	0.91		0.89		0.83		0.82
Benefit to mepolizumab (omalizumab = 2.5)– (mepolizumab = 1.5) = 1	0.00, 100 (vague)	5	>0.99		>0.99		0.98		0.97
	0.00, 0.347 (no difference)	6	>0.99		>0.99		0.99		0.98
	0.34, 0.347 (skeptical for mepolizumab)	7	>0.99		>0.99		0.98		0.97
	– 0.51, 0.47 (optimistic for mepolizumab)	8	>0.99		>0.99		0.99		0.99
Benefit to omalizumab (omalizumab = 2.5)– (mepolizumab = 3.5) = –1	0.00, 100 (vague)	9	0.14		0.21		0.33		0.32
	0.00, 0.347 (no difference)	10	0.14		0.2		0.36		0.39
	0.34, 0.347 (skeptical for mepolizumab)	11	0.14		0.16		0.3		0.32
	– 0.51, 0.47 (optimistic for mepolizumab)	12	0.14		0.22		0.44		0.47

* For selection of these values see “Plausible priors” section in the Supplementary file. The table provides results of the average posterior probability under 12 different scenarios based on three different treatment effects: when mepolizumab is noninferior, superior, and inferior to omalizumab. Each treatment effect assumes an optimistic, no difference, and skeptical prior under the assumption of a Poisson distribution and also under the assumption of overdispersion in outcome using a negative binomial regression analysis model.

## Data Availability

Only simulated data and the code and parameters are in the paper so people can generate.

## What is new?

### Key findings

- A Bayesian framework can enable treatment evaluation against a credible non-inferiority margin.
- Demonstrating ‘probability of non-inferiority’ for differing scenarios can be used instead of a sample size calculation.

### What this adds to what is known?

- Use of wide non-inferior margins is common and can result in harmful conclusions.

### What is the implication, what should change now?

- We encourage a shift away from sizing trials on unrealistic non-inferiority margins.
