# Supplementary material for: A Bayesian framework to evaluate non-inferiority in randomized controlled trials of uncommon conditions
Source: J Clin Epidemiol. Author manuscript; Available in PMC 2026 Aug 5. (PMC7619311; doi:10.1016/j.jclinepi.2025.112002)
Supplement: Supplementary file 1 [file EMS213770-supplement-Supplementary_File_1_.docx]

# TREAT Trial case study

Ineffective inhaler technique or not taking prescribed medication can be the cause of uncontrolled asthma, but there is an important severe disease sub-group of children who, despite taking optimal therapy effectively, remain uncontrolled, placing them at high risk of poor outcomes. The uncommon condition in this example is children with Severe Therapy Resistant Asthma (STRA). The TREAT trial aims to examine the non-inferiority of mepolizumab against omalizumab (an established treatment for STRA) as a means to obtain indirect evidence of efficacy for mepolizumab.

Funding was sought from NIHR Efficacy and Mechanism Evaluation (EME) programme. A previous iteration of the application included classical efficient trial design features using a placebo-controlled crossover trial design with repeated measurements on a continuous outcome (a validated severity score based on treatment use and symptoms). The programme was supportive of the research question but initially rejected the application and provided feedback:1) to change the primary outcome to rate of asthma exacerbations (an outcome that has less information in it compared to a continuous outcome); 2) not to use a crossover design; 3) not to include a placebo control due to ethical considerations (dictating a non-inferiority comparison). These design recommendations set by the funding committee removed all efficient design features from the trial (as recommended in Parmar et al.[1]) As sample size was highly restricted this presented a problem to powering a new NI trial using a traditional significance-hypothesis testing approach.

As a response to the feedback we proposed a multicentre, randomised (1:1), parallel arm, NI trial of mepolizumab against omalizumab with asthma exacerbation rate over 12 months as the primary outcome.[2] Instead of powering the study based on a significance-hypothesis test, we used the proposed framework to demonstrate the potential value of the trial using a Bayesian approach.

The trial would be worth undertaking if, with the proposed sample size, the posterior probabilities for non-inferiority were adequately high under the scenarios where mepolizumab is non-inferior or superior to omalizumab, and reassuringly low for the scenarios where mepolizumab is inferior. These results would demonstrate that the trial, regardless of the true treatment effect, could inform future policy and prescribing decisions. The following sets out our approach to the redesign and justification to reviewers.

# Generating TREAT Trial’s simulation set

1) Data Generation and Analysis Model Family: A Poisson regression model with an offset for time was chosen as the primary analysis model to compare 12-month asthma exacerbation rate between arms. Gaussian prior distributions were used for two model parameters, α and β, representing the log exacerbation rate in the established treatment arm, and change in log exacerbation rate between treatment arms respectively.

log(μ_i_) = α + $\delta$ x_i_ + log(t_i_)

α: log exacerbation rate in omalizumab arm, $e^{\alpha}$= rate in omalizumab

$\delta$, : difference in log exacerbation rate between treatment arms, $e^{\delta}$= ratio of rate between treatment arms, Incidence rate ratio (IRR)

x_i_: treatment arm (0= omalizumab, 1= mepolizumab) for participant i

t_i_: follow-up time for participant i

As overdispersion in the outcome is possible we also examined a negative binomial regression model for the analysis model. Data for overdispersion in simulations were generated by drawing observations from a Poisson distribution with appropriate mean value and adding a random draw from a gamma distribution using a shape parameter of 1 and a scale parameter of 0.6 (estimated from clinical audit data).

## 2)Trial Sample size

To estimate the maximum sample size, feasibility assessments were undertaken across all collaborating UK specialist clinics (n=11). Over a three-year recruitment period it was estimated that around 500 children would be identified with severe uncontrolled asthma and recruited to the run-in period for the trial, from which approximately 150 would subsequently be diagnosed with STRA and to be randomised to trial treatment. With an anticipated 15% loss to follow-up we estimated we could achieve 130 children with 12-month outcome data.

## 3) Scenario:

An exacerbation rate of 2.5 per year for the established treatment (omalizumab) was estimated based on a combination of the eligibility criteria and published evidence for the efficacy of omalizumab i.e.to be eligible for the trial, participants must experience a minimum of four exacerbations in the year prior, and it is expected this may average 5 per year in the recruited population. Omalizumab has been shown to reduce the exacerbation rate by approximately 50%.[3, 4]

For the new treatment (mepolizumab) the mean exacerbation rate was specified with reference to omalizumab and having chosen that a change in 1 exacerbation per year was a difference that would represent a superior or inferior treatment. The three potential outcomes evaluated were:

a. Efficacy of new = established: mepolizumab mean exacerbation rate of 2.5
b. Efficacy of new > established: mepolizumab mean exacerbation rate of 1.5
c. Efficacy of new < established: mepolizumab mean exacerbation rate of 3.5

The 12 scenarios are constructed based on examining 4 priors on each potential outcome and are listed in Table 1.

## 4) Primary Non-Inferiority (NI) Margin

A NI margin of 0.5 exacerbations per year was chosen as a primary NI margin after discussion with clinicians and parents. A reduction of one exacerbation per year was reasoned to be the minimum benefit required given 12 months of injections with either treatment, the primary non-inferiority margin was taken to be half the minimum benefit required. The final trial analysis will also calculate results for NI margins from 0.1 to 1.

## 5) Plausible Priors

The prior distributions for the analysis were unspecified at the design stage (an expert consensus elicitation was planned after funding received). In the absence of any information a Gaussian distribution was chosen for the prior distributions on the log exacerbation rate in omalizumab arm, and the change in log exacerbation rate between arms (treatment effect). We discuss the specification of the parameters of these priors in more detail below.

α ~ N(μ_α_, $\sigma_{\alpha}^{2}$) prior distribution for the log exacerbation rate in omalizumab arm

$\delta$ ~ N(μ_δ_ , $\sigma_{\delta}^{2}$) prior distribution for change in log exacerbation rate between arms

For each of the three informative Gaussian priors on the treatment effect β, a location (mean) and standard deviation (SD) need to be selected. There are multiple ways this can be approached. We chose the location with reference to what was considered to be a minimal clinical important shift in the outcome and selected the SD with consideration to the no-difference scenario. We step through how we selected parameter values below:

i) **Define the SD of the** $\boldsymbol{\delta}$ **priors for optimistic, sceptical and no difference scenarios**

*We selected the SD of the prior distribution on* $\delta$ *by first considering the ‘no difference’ prior. The value of 0.347 was chosen by selecting a plausible upper bound for the 95% confidence interval of the IRR (exp(*$\delta$*)). A doubling of the IRR was judged a plausible value for which there would be a very low probability (0.025) that the true IRR would be larger than this if there really was no difference between treatments. The SD was estimated from:*

*[Ln(IRR)_upper 95%CI_ – Ln(IRR)_mean_] / 2, where the ‘no difference’ IRR _mean_ = 1.*

*The estimated SD is calculated by [Ln(2)-Ln(1)]/2 = 0.347*

*This SD was used for all three informative priors.*

ii) **Define the mean of the** $\boldsymbol{\delta}$ **priors for optimistic, sceptical and no difference scenarios**
    a. **No difference prior mean:***12-month exacerbation rate is assumed to be 2.5 per year on omalizumab and mepolizumab. Ln(IRR) = Ln (2.5/2.5) = 0*
    b. **Optimistic prior mean:** *A minimally clinical important difference of 1 exacerbation per year is used as an important shift in the distribution, under the optimistic scenario exacerbations will reduce from 2.5 to 1.5 on mepolizumab. Ln(IRR) = Ln(1.5/2.5)= -0.51.*
    c. **Sceptical prior mean:** *As in b., under the sceptical scenario exacerbations will increase to 3.5 on mepolizumab. Ln(IRR) IRR = 3.5/2.5, Ln(3.5/2.5)= 0.34.*

*The prior distributions can be seen in Supp. Figure 1*

iii) **Define** $\boldsymbol{\delta}$ **for the vague prior scenario:** *Chosen to be centred on no effect but with large SD, we selected mean = 0 and SD = 100*
iv) **Define the mean of the prior on α (log exacerbation rate) for all scenarios:**

*An uncontroversial choice for the mean is to base it on the expected exacerbation rate in the omalizumab arm of 2.5,* Ln (2.5) = 0.916.

v) **Define the SD of the α prior for all scenarios:**

*A SD of 0.347 was used as this was considered a reasonable choice for uncertainty in the log mean exacerbation rate based on published data for this population and matches the SD for the SD chosen for the prior on β.*

vi) ***Define the test threshold to calculate the posterior probability of non-inferiority***
    a. *NI Margin of 0.5 exacerbations will give a test threshold in the Log IRR of Log(3.0/2.5)=Log(1,2) which we used to calculate the posterior probability of non-inferiority (i.e. Pr(*$\delta$ *<* $\delta^{*}$*)*

For MCMC sampling we have specified a burn-in of 500 samples, thinning every 4 to reduce auto-correlation and generate 5,000 MCMC samples. For each of the 12 scenarios we generated 1000 simulations.

Example Stata code for a single scenario example can be found in Supplementary section 2. We summarised simulation results by calculating the **average posterior probability of being non-inferior for a 0.5 NI margin**. We present histograms to examine the distribution of the posterior probabilities.

We repeated simulations for 75% of the sample size for the purpose of assessing the impact of including fewer participants.

Supp. Table 1 displaying prior location and standard deviation.

**Supp. Figure 1:** Showing the prior distributions (optimistic, no difference, sceptical) used with reference to the non-inferiority margin on the on the natural logarithm scale

**Supp. Figure 2:** The results displaying the influence of the priors.

The TREAT trial design simulations showing the posterior mean and 95% Credible Interval under the three scenarios (favouring mepolizumab, favouring omalizumab, no treatment effect) with four different priors (vague, no difference, sceptical for mepolizumab, optimistic for mepolizumab) scenarios 1 to 12, by two different sample size (130 and 98) and assumptions for outcome distribution ( Poisson and over dispersed)

a) Sample size of 130 assuming outcome follows a Poisson distribution

b) Sample size of 98 assuming outcome follows a Poisson distribution

c) Sample size of 130 assuming outcome is over dispersed (negative binomial distribution)

d) Sample size of 98 assuming outcome is over dispersed ( negative binomial distribution)

Note that the impact of the priors in minimal with a sample size of 130 and under the strong assumption the outcome follows a Posson Distribution. With a smaller sample size of 98 then there is slight influence but the influence greater if the outcome is dispersed, especially for the smaller sample size.

**Supp. Table 2:** Results of the simulations

Poisson with sample size n=130

Poisson with sample size n=98

Over dispersed with sample size n=130

Over dispersed with sample size n=98

**REFERENCES**

1.	Parmar, M.K., M.R. Sydes, and T.P. Morris, *How do you design randomised trials for smaller populations? A framework.* BMC Med, 2016. **14**(1): p. 183.

2.	S, S. *Treating severe paediatric asthma: the TREAT trial*. 2020 [cited 2020 12/12/2020]; Available from: <http://www.isrctn.com/ISRCTN12109108>.

3.	Lanier, B., et al., *Omalizumab for the treatment of exacerbations in children with inadequately controlled allergic (IgE-mediated) asthma.* J Allergy Clin Immunol, 2009. **124**(6): p. 1210-6.

4.	Kulus, M., et al., *Omalizumab in children with inadequately controlled severe allergic (IgE-mediated) asthma.* Curr Med Res Opin, 2010. **26**(6): p. 1285-93.
