# Supplementary material for: A Bayesian framework to evaluate non-inferiority in randomized controlled trials of uncommon conditions
Source: J Clin Epidemiol. Author manuscript; Available in PMC 2026 Aug 5. (PMC7619311; doi:10.1016/j.jclinepi.2025.112002)
Supplement: Supplementary file 2 [file EMS213770-supplement-Supplementary_File_2.docx]

**TREAT Trial Simulation code in Stats for one scenario**

This example will demonstrate how data can be generated and model parameters be estimated. We will use a trial example where the maximum feasible sample size to recruit is 150 participants, with 15% loss to follow-up so data will be generated for 130 individuals. The outcome is a count of the number of expected exacerbations per year.

In this example we will compare the two treatments when the new treatment if superior to the established treatment. In simulations you would normally set up a range of scenarios and get estimates for each one, here we will start with just one.

For this scenario, we will set the prior distributions as uninformative (i.e. wide distributions) and generate data. The full list of parameters for this scenario is below.

1. **Parameters for Scenario**

- Prior for 𝛽 = Uninformative = $\sim N(0,{100}^{2})$
- Prior for 𝛼 = Uninformative = $\sim N(0,{100}^{2})$
- Mean exacerbations in Established arm (Omalizumab) = 2.5
- Mean exacerbations in new Active arm (Mepolizumab) = 1.5

2. **Generate the Data (Likelihood)**

The first step is to generate the data. The maximum feasible sample size is 150. Assuming a 15% loss to follow up we generate observations for 130 participants:

- The first 65 are in ‘trt’ == 0 which is the established treatment. (Omalizumab)
- The second 65 are in ‘trt’ == 1 which is our new active treatment ( Mepolizumab)

For each treatment arm we randomly generate values from a suitable outcome distribution. For our example as the outcome is the number of exacerbations per year we use a Poisson distribution. We sample Mepolizumab from a distribution with mean 1.5, and Omalizumab from a distribution with a mean of 2.5.

```
/* --- bayes model example for Sample Size --- */
drop _all
qui {
set obs 130 // the number of participants in your trial
gen id    = _n
gen trt   = (_n > (_N/2))
gen y     = rpoisson(2.5) if trt == 0 // established arm with mean 2.5
replace y = rpoisson(1.5)  if trt == 1 //new arm with mean 3.5
}

list in 1/4
```

```
list in 1/4 if trt == 0 //look at the first 4 observations in established arm

     +--------------+
     | id   trt   y |
     |--------------|
  1. |  1     0   7 |
  2. |  2     0   0 |
  3. |  3     0   3 |
  4. |  4     0   0 |
     +--------------+

gsort -trt // sort new arm to top of dataset
list in 1/4 // now list will show new arm data

     +---------------+
     |  id   trt   y |
     |---------------|
  1. | 108     1   0 |
  2. | 130     1   1 |
  3. | 128     1   0 |
  4. | 106     1   0 |
     +---------------+
```

3. **Setup Priors**

We next specify the mean and SD for the prior distributions.

- prior for 𝛼 is the Log(Exacerbation rate in Omalizumab)
- prior for β is the Log (IRR)

Choosing these parameters are discussed in full in Supp1. For this scenario we use vague priors centred on 0 with SD of 100 (variance of 10000).

Note that this code section need to be run in Stata together with section 4 in order for the macros to be stored and called correctly by the Bayesian model.

```
* Established Arm Parameters
local logodds_control =  0
local logodds_var_control = 10000
local control_prior "`logodds_control' , `logodds_var_control'" // macro for bayes model

* New Active Arm Parameters
local beta_logodds = 0
local beta_var      = 10000
local prior "`beta_logodds', `beta_var'" // macro for bayes model
```

4. **Performing the Bayesian Sampling**

Once the prior and Likelihood have been defined the analysis model can be run. In the Bayesian command (bayes) we have specified a burn-in of 500 samples, thinning every 4 to reduce auto-correlation and generate 5,000 MCMC samples.

We then calculate the probability of non-inferiority using the NI margin, on the IRR scale this is < 1.2 (using bayes test)

```
* Bayes Model
bayes, burn(500) thinning(4) mcmcs(5000) /// MCMC Sampling Parameters
        prior({y:trt}, normal(`prior')) prior({y:_cons}, normal(`control_prior')) /// Priors call Macros Above
        : glm y trt, family(Poisson) link(log) //  Model to use


local mtest = log(1.2) // Convert the IRR to Log (same scale as model)
bayestest interval {y:trt}, upper(`mtest')
```

```
note: discarding every 3 sample observations; using observations 1,5,9,....

Model summary
------------------------------------------------------------------------------
Likelihood:
  y ~ poisson(xb_y)

Prior:
  {y:trt _cons} ~ normal(0,10000)                                          (1)
------------------------------------------------------------------------------
(1) Parameters are elements of the linear form xb_y.

Bayesian Poisson regression                      MCMC iterations  =     20,497
Random-walk Metropolis–Hastings sampling         Burn-in          =        500
                                                 MCMC sample size =      5,000
                                                 Number of obs    =        130
                                                 Acceptance rate  =       .167
                                                 Efficiency:  min =      .3229
                                                              avg =      .3232
Log marginal-likelihood = -227.72308                          max =      .3236

------------------------------------------------------------------------------
             |                                                Equal-tailed
           y |      Mean   Std. dev.     MCSE     Median  [95% cred. interval]
-------------+----------------------------------------------------------------
         trt | -.5389987   .1342129    .00334  -.5405363  -.8054476  -.2814386
       _cons |   .826379   .0798047   .001984   .8281536    .666924   .9797136
------------------------------------------------------------------------------
Note: Adaptation continues during simulation.

Interval tests     MCMC sample size =     5,000

       prob1 : {y:trt} < .1823215567939546

-----------------------------------------------
             |      Mean    Std. dev.      MCSE
-------------+---------------------------------
       prob1 |         1     0.00000          0
-----------------------------------------------
```

The mean difference in the log exacerbation rate between treatment arms is -0.5389 ( see figure 2 below for depiction of the posterior probability distribution. We then want to calculate what proportion of the posterior distribution lies below the NI margin = Log(1.2) = 0.1823

As this is the scenario where the new treatment is superior to the established treatment the then probability of non-inferiority is very high. From the above output we can see this reported a prob1 = 1, but we would record this a >0.99

# 5. Diagnostics

In order to check the model convergence we generate diagnostic plots. In the plots we want to see that our assumptions are met, i.e. that autocorrelation is reasonably low and the trace plot has a clear mean across iterations with no trend.

bayesgraph diagnostics {y:_cons}, histopts(normal) title("Alpha Term") // For Alpha Term

Figure 1

bayesgraph diagnostics {y:trt}, histopts(normal) title("Beta Term") // For Beta Term

Figure 2

# 6. Create a Loop

The above will create a single set of posterior estimates under the Likelihood/Priors of this scenarios. However the sample size is small so there is uncertainty in the random draws when generating the data that may have influenced the estimates. Therefore, it is good practice to repeat this process and generate more draws from the Poisson distribution and re-run the Bayes model in order to get a range of the plausible posterior estimates. To do this we need to put the code above into a loop and save the outputs to a new stata file, to do this we will use the postfile command.

```
* --- Create a file to save results to
cap postclose simResults // in case it's open
postfile simResults rep cprior_m cprior_var aprior_m aprior_var ///
		beta betaLCI betaUCI postprob using simResults , replace // each word in list is a variable name (e.g. rep or cprior_m)

* --- In this example we aren't varying Priors so this code can stay outside the loop
* Establish Arm Parameters
local logodds_control =  0
local logodds_var_control = 10000
local logodds_var_control = 0.347
local control_prior "`logodds_control' , `logodds_var_control'" // macro for bayes model

* New Active Arm Parameters
local beta_logodds = 0
local beta_var      = 10000
local prior "`beta_logodds', `beta_var'" // macro for bayes model
local beta_var      = 0.80
local prior "`beta_logodds', `beta_var" // macro for bayes model


* --- Perform the Simulation 10 times
forvalues r = 1/10 {

    * Generate Data
    drop _all
    qui { // To generate without any output
        set obs 130
        gen id    = _n
        gen trt   = (_n > (_N/2))
        gen y     = rpoisson(2.5) if trt == 0
        replace y = rpoisson(1.5)  if trt == 1
    }

    * Run Bayes Model
    bayes, burn(500) thinning(4) mcmcs(5000) /// Bayes Options (burn-in, thinning and MCMC samples)
            prior({y:trt}, normal(`prior')) prior({y:_cons}, normal(`control_prior')) /// Call the priors saved to local macros above
            : glm y trt, family(Poisson) link(log) //  Specify the analysis model used to analyse the outcome

    * Save Posterior Estimates to local macros so can be posted to file
    mat define TE=e(mean)
    local beta = TE[1,1]
    mat define CI=e(cri)
    local betaLCI = CI[1,1]
    local betaUCI = CI[2,1]

    * Save test margin probability so this can be posted to file
    local mtest = log(1.2) // Convert the IRR to Log (same scale as model)
    bayestest interval {y:trt}, upper(`mtest')
    mat define probs=r(summary)
	local pp = probs[1,1]

    * Post to file called simResults
    post simResults (`r') (`logodds_control') (`logodds_var_control') (`beta_logodds') (`beta_var') ///
            (`beta') (`betaLCI') (`betaUCI') (`pp')
}

* Now finished close the file to look at
cap postclose simResults
```

# 7. Repeat for Other Scenarios

These steps should then be repeated for the other scenarios where the prior distributions or the likelihood are varied.
